# Ligneous conjunctivitis: Fresh-frozen plasma and heparin use intra-and postoperatively, a report of two cases

**DOI:** 10.5935/0004-2749.2022-0288

**Published:** 2024-03-05

**Authors:** Sabela Costa Guerra Barreto de Almeida, Patrícia Maria Fernandes Marback

**Affiliations:** 1 Serviço de Oftalmologia, Hospital Universitário Professor Edgard Santos, Universidade Federal da Bahia, Salvador, BA, Brazil

**Keywords:** Conjunctivitis, Plasm, Heparin, Plasminogen, Ophthalmic solutions, Administration, ophthalmic

## Abstract

Ligneous conjunctivitis is a rare chronic form of recurrent membranous inflammation and plasminogen deficiency. Ocular manifestations may be associated with sites other than mucous membranes, such as the oral cavity, internal ear, respiratory, genitals, and kidney. Treatment is extremely difficult because of the lack of topic plasminogen drops, and a high volume is required for systemic supplementation. This report aimed to present two patients with ligneous conjunctivitis treated with membrane excision, topical fresh-frozen plasma, and heparin intra-, and postoperatively. No recurrence was found in the ligneous membrane in the 12-month follow-up. The use of topical fresh-frozen plasma and heparin after membrane excision could be effective to avoid recurrence.

## INTRODUCTION

Ligneous conjunctivitis (LC) is a rare form of recurrent membranous inflammation^(1)^. It is associated with congenital plasminogen deficiency, which is a rare hereditary fibrinolytic system autosomal recessive disorder^(2)^. Ocular manifestations may be associated with systemic involvement of other mucous membrane^(2,3)^. It usually occurs during infancy and childhood, with slight female predominance^(2)^. In addition, <20% occur in the fourth and fifth decades of life^(4)^. Disease onset is reported at a mean age of 13-16 years^(2)^.

LC treatment is difficult because of the lack of both topical plasminogen and systemic recommendations for substitution therapy^(5,6)^. So far, no consensus has been established on the best therapeutic approach. Some authors advocated topical fresh-frozen plasma (FFP) and heparin use, showing good initial results^(4,5,7)^.

Herein, we describe two cases of LC treated with subconjunctival and topical FFP and heparin after simple membrane excision, showing good results in the 12-month follow-up period.

## CASES REPORT

On hematological evaluation, both patients presented recurrent chronic conjunctival membranes and plasminogen deficiency. The treatment protocol consisted of simple membrane excision, FFP, heparin, and combined eye drops 6/6 h, as described in chart 1.

**Chart 1:** Treatment protocol

FFP preparation	The plasma was displaced by local blood bank. Packaged in 1-mL sterile syringes, frozen at -40°C One syringe disposable each day, refrigerated at 4°C during use
Surgery	Simple membrane excision with minimum cauterization, local anesthesia FFP, 1.0 mL subconjunctival Heparin 5000 UI/mL drops during the procedure
Post operative	Combined eye drops 6/6 h (dexamethasone 0.1% + neomycin and polymycin B) for 7 days Loteprednol, 6/6 h weekly weaned off Heparin 5000 UI/mL 2/2 h until re-epithelialization, stored at room temperature (stored at sterile eye drop bottle) FFP drops 2/2 h for 3 days, 3/3 h for 30 days

FFP: fresh frozen plasma

## CASE 1

**Figure 1:**
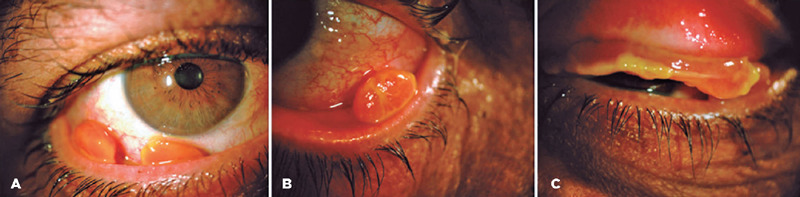
Case 1: Biomicroscopy on admission. (A) and (B) lower tarsal pink, granulomatous aspect, pedunculated masses. (C) OS upper tarsal, thick, and hard membrane.

A 46-year-old male patient was referred for the treatment of surgical and steroid refractory conjunctival membranes associated with recurrent purulent conjunctivitis in both eyes for the last 2 years. He was under colitis investigation following a 2-month diarrhea report. All teeth were lost in adolescence because of alveolitis of unknown cause. His corrected visual acuity (VA) was 20/30 in the right eye (OD) and 20/80 in the left eye (OS). Pseudoptosis caused by a true membrane on OS upper tarsus was identified. Biomicroscopy of both eyes revealed lower tarsal pink, granulomatous aspect, pedunculated nodules, and OS upper tarsal showed a thick and hard sessile membrane (Figure 1).

**Figure 2:**
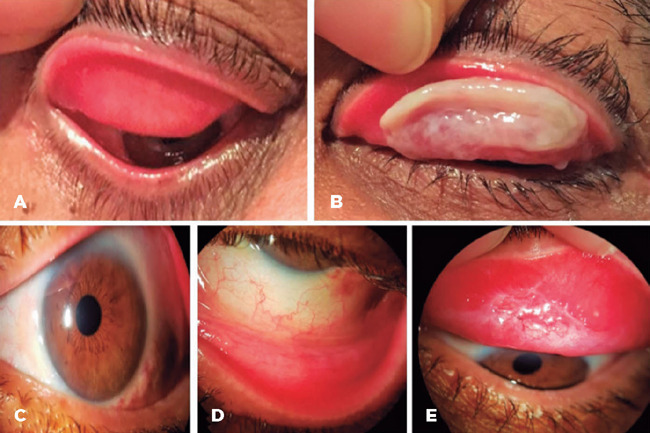
Case 1, 3 years after admission: (A) OD without membrane; (B) OS upper tarsal ligneous membrane; (C–E) OS without recurrence 12 months after membrane excision plus topical FFP and heparin.

Reduced plasminogen values of 4.75 mg/dL (reference value [RV] 6.00-25.00) were detected. Owing to a history of previous multiple recurrences, a new membrane excision was scheduled, and this time with subsequent use of topical serum from a normal plasminogen donor, the patient’s wife. No recurrence occurred in OD. In OS, a new recurrence was treated with topical cyclosporine, followed by topical tacrolimus, with no improvement (Figure 2B).

Three years after the last excision, in the absence of spontaneous resolution, OS membrane excision was scheduled using FFP and heparin, as described above. No membrane recurrences were noted in the 12-month follow-up (Figure 2C-2E).

## CASE 2

**Figure 3:**
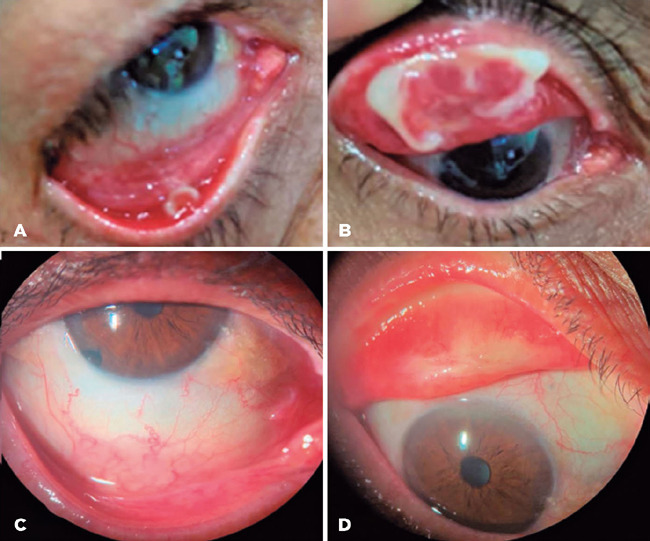
Case 2: before and after FFP + heparin. (A) OD, lower tarsal membrane; (B) OD, upper tarsal membrane; (C) and (D) OD without recurrence 12 months after membrane excision plus topical FFP and heparin.

A 70 years-old female patient was referred for the evaluation of chronic membranous conjunctivitis in the OD refractory to clinical treatment and three surgical removal attempts on the last 12 months. She reported the first manifestation at age of 2, with spontaneous improvement. Her left eye was enucleated 50 years ago; however, she could not specify the reason. She had a medical history of asthma and systemic arterial hypertension. On examination, corrected VA was OD 20/50, and biomicroscopy revealed a thick, hard consistency upper tarsal membrane and a smaller one at the lower tarsal conjunctiva (Figure 3A and 3B). No changes were observed on the left anophthalmic socket. Serum plasminogen activity was 43% (RV: 75%-100%), confirming the suspicion of LC, and OD membrane excision with the use of FFP and heparin was arranged following the described protocol. The patient evolved without new lesions in a 12-month follow-up (Figure 3C and 3D).

## DISCUSSION

LC is a rare disease associated with systemic plasminogen deficiency. Inherited plasminogen (PLG) deficiency in humans has two types: true PLG deficiency, type I or hypoplasminogenemia, and dysplasminogenemia or type II ^(8)^. In the former, both immunoreactive PLG level, and functional activity are reduced, wheras the latter shows a normal or slightly reduced level of immunoreactive PLG, although functional activity is significantly decreased^(8)^. LC is the most common clinical manifestation and is characterized by recurrent conjunctival membranes^(1,2,8)^.

Despite some reports of spontaneous membrane resolution in LC, both of our patients showed persistent, refractory, and symptomatic membranes. These lesions typically present in childhood; however, according to literature, they can occur at any age^(8)^.

This rare condition can be distressful for many reasons, including late diagnosis (both patients here reported) and persistent symptoms, and once the diagnosis is confirmed, no treatment is easily available. Furthermore, symptoms may be disabling. Our patients reported mild to moderate pain due to the membrane contact with ocular surface and frequent conjunctival discharge, interfering with daily activities, and social interactions.

Purified PLG replacement therapy is a promising approach without severe adverse events^(6,9,10)^. However, it is not available in most health centers and may be cost limiting in developing countries. Topical and systemic FFP are described as alternatives^(1,4,9)^. Systemic replacement requires high-volume intravenous administration, which can be associated with collateral side effects. The isolated use of topical FFP, with, or without membrane excision, showed good results in local disease, control^(1,4)^. In addition, the topical use of heparin is described as isolated or associated with cyclosporine eye drops^(5,7)^. The two cases reported herein had a long history of symptomatic persisted membranes; thus, we opted for surgical excision followed by topical replacement therapy^(1,4,5,9)^.

The stability of FFP was an issue of the treatment protocol. The eye clinic is part of a general hospital, and we could use hemotherapy facilities to fractionate and store the plasma. This case report shows that LC remains challenging concerning early diagnosis and treatment. Topical FFP and heparin use was associated with better disease control with a minimally invasive procedure, had a low cost, and could be effective in preventing new membrane recurrence in the 12-month follow-up.
